# A Household Serosurvey to Estimate the Magnitude of a Dengue Outbreak in Mombasa, Kenya, 2013

**DOI:** 10.1371/journal.pntd.0003733

**Published:** 2015-04-29

**Authors:** Esther M. Ellis, John C. Neatherlin, Mark Delorey, Melvin Ochieng, Abdinoor Haji Mohamed, Daniel Ondari Mogeni, Elizabeth Hunsperger, Shem Patta, Stella Gikunju, Lilian Waiboic, Barry Fields, Victor Ofula, Samson Limbaso Konongoi, Brenda Torres-Velasquez, Nina Marano, Rosemary Sang, Harold S. Margolis, Joel M. Montgomery, Kay M. Tomashek

**Affiliations:** 1 Dengue Branch, Division of Vector-Borne Diseases, Centers for Disease Control and Prevention, San Juan, Puerto Rico; 2 Division of Global Health Protection, Center for Global Health, Centers for Disease Control and Prevention, Nairobi, Kenya; 3 Division of Vector-Borne Diseases, Centers for Disease Control and Prevention, Fort Collins, Colorado, United States of America; 4 Center for Global Health Research, Kenya Medical Research Institute, Nairobi, Kenya; 5 Ministry of Health, Mombasa, Kenya; 6 Centre for Virus Research, Kenya Medical Research Institute, Nairobi, Kenya; U.S. Naval Medical Research Unit No. 2, INDONESIA

## Abstract

Dengue appears to be endemic in Africa with a number of reported outbreaks. In February 2013, several individuals with dengue-like illnesses and negative malaria blood smears were identified in Mombasa, Kenya. Dengue was laboratory confirmed and an investigation was conducted to estimate the magnitude of local transmission including a serologic survey to determine incident dengue virus (DENV) infections. Consenting household members provided serum and were questioned regarding exposures and medical history. RT-PCR was used to identify current DENV infections and IgM anti-DENV ELISA to identify recent infections. Of 1,500 participants from 701 households, 210 (13%) had evidence of current or recent DENV infection. Among those infected, 93 (44%) reported fever in the past month. Most (68, 73%) febrile infected participants were seen by a clinician and all but one of 32 participants who reportedly received a diagnosis were clinically diagnosed as having malaria. Having open windows at night (OR = 2.3; CI: 1.1–4.8), not using daily mosquito repellent (OR = 1.6; CI: 1.0–2.8), and recent travel outside of Kenya (OR = 2.5; CI: 1.1–5.4) were associated with increased risk of DENV infection. This survey provided a robust measure of incident DENV infections in a setting where cases were often unrecognized and misdiagnosed.

## Author Summary

Dengue appears to be endemic in Africa with a number of reported outbreaks. In February 2013, several individuals with dengue-like illnesses and negative malaria blood smears were identified in Mombasa, Kenya. Dengue was laboratory confirmed and an investigation was conducted to estimate the magnitude of local transmission including a serologic survey to determine incident dengue virus (DENV) infections. Consenting household members provided serum and were questioned regarding exposures and medical history. RT-PCR was used to identify current DENV infections and IgM anti-DENV ELISA to identify recent infections. Of 1,500 participants, 13% had evidence of current or recent DENV infection. Among those infected, 44% reported fever in the past month. Most (73%) febrile infected participants were seen by a clinician, and all but one of the 32 participants who reportedly received a diagnosis were clinically diagnosed as having malaria. Having open windows at night, not using daily mosquito repellent, and recent travel outside of Kenya were associated with increased risk of DENV infection. This survey provided a robust measure of incident DENV infections in a setting where cases were often unrecognized and misdiagnosed.

## Introduction

Dengue is a mosquito-borne acute febrile illness caused by one of four dengue viruses (DENV-1-4). It is endemic throughout the tropics and sub-tropics [1,2] where more than 40% of the world’s population is at risk and approximately 400 million DENV infections are estimated to have occurred in 2010 alone [3]. While the majority of infections are asymptomatic or result in a mild febrile illness, about one-quarter of infected people have signs and symptoms consistent with dengue or severe dengue [3,4]. Infection with any DENV type may result in dengue, an illness characterized by fever, headache, retro-orbital eye pain, myalgia, arthralgia, minor hemorrhagic manifestations, and rash [4]. Although most dengue patients will recover within one week, 5 to 10% of patients in endemic areas will progress to severe dengue, which includes dengue hemorrhagic fever (DHF) and dengue shock syndrome (DSS), and is characterized by thrombocytopenia, plasma leakage due to increased vascular permeability, severe organ involvement, and/or clinically significant bleeding [5–8]. Of severe dengue patients, 0.1–10% will not survive, which is dictated in part by the timing and quality of medical care. Medical care with appropriate intravascular volume repletion and supportive intensive care has been shown to lower mortality associated with severe dengue [4,9,10].

Dengue is likely endemic throughout much of Africa where the mosquito vector is widely present [3]. However, from 1960–2010, only 22 of 52 African countries reported sporadic cases or dengue outbreaks because of a general lack of awareness of the disease, surveillance or availability of diagnostic testing [11]. Although recent estimates suggest a substantial disease burden in Africa with an estimated 65 million annual DENV infections, they also demonstrated the uncertainty of the geographic distribution of DENV transmission and disease [3].

Reports of dengue occurrence from Africa constitute only a small percentage (<4%) of all globally reported disease and only a single country in Africa has reported cases to the World Health Organization (WHO) [3]. In countries such as Kenya, where no dengue cases were reported from 1986 to 2013, population-based studies are needed to better understand the epidemiology and true incidence of disease and infection [11–18].

In February–March 2013, 71 patients with an acute febrile illness and negative malaria blood smears were identified in Mombasa, Kenya, and reported to the National Ministry of Health (MOH). Nearly half (46%) were confirmed to have an acute DENV infection by real-time, reverse transcriptase-polymerase chain reaction (RT-PCR). The Kenya MOH in collaboration with the Centers for Disease Control and Prevention (CDC) Center for Global Health, CDC Dengue Branch, and Kenya Medical Research Institute (KEMRI) responded by conducting an outbreak investigation that included establishing enhanced hospital surveillance in Mombasa followed by a serological survey of the population in Tudor district, an area of initial case detection, in order to provide an estimate of local dengue incidence and identify risk factors for DENV infection.

## Methods

### Study Area

Mombasa, the second largest city in Kenya, has an approximate population of 939,370 residents and is a major port and international tourist destination in East Africa [5]. Hospital surveillance was established in Mombasa, which is comprised of six constituencies and thirty districts. Tudor district of Mombasa was selected as the site for a seroincidence survey because initial dengue cases were detected from area residents. The Tudor district is located at 4°2′22″S 39°39′48″E and has a population of approximately 10,000 residents [5].

### Hospital Surveillance

Hospital surveillance activities, which included active case finding and chart reviews, were performed to follow temporal dengue trends in Mombasa and determine the effectiveness of control activities. Enhanced surveillance for dengue-like acute febrile illnesses was initiated at seven hospitals: three public (Tudor Hospital, Coast Province General Hospital, Port Reitz District Hospital) and four private (Aga Khan Hospital, Mombasa Hospital, Pandya Memorial Hospital, Bomu Hospital). Physicians, nurses, and laboratory staff at these hospitals were trained in clinical recognition and management of dengue, and asked to submit serum specimens and patient information from suspected dengue cases. Suspected case-patients were defined as patients with an acute febrile illness (i.e., fever ≥38°C for ≤7 days), absence of cough, a negative malaria blood smear, and at least one additional sign or symptom consistent with dengue including retro-orbital pain, headache, rash, myalgia, arthralgia, leucopenia, or a hemorrhagic manifestation. Dengue diagnostic testing was performed as described below (see Laboratory Diagnosis).

### Seroincidence Survey

A sample size of 1,500 individuals was calculated based on an assumed prevalence of recent DENV infection of approximately 4%, an absolute error of +/- 3%, and a design effect of two to account for intra-household clustering. Assuming that an average of 1 to 2 persons per participating household would consent to provide a blood specimen, 986 households were randomly selected for enrollment in 5 geographic areas within Tudor district. The areas were selected based on their population density, housing type, and socioeconomic diversity. The primary sampling unit for the household survey was a housing plot. In four of the five areas, housing plots were enumerated using a combination of a local and Google Earth map (Google, Inc.), and a random number generator (R v2.15.1) was used to select housing plots for inclusion. In one area, each plot contained a single house and no subsampling was performed. In three areas, multiple households were located on a single plot and random sampling of households was carried out by enumeration of households with subset selection using a random digits table carried by the field teams. The fifth area contained high density housing in which housing plots could not be identified; field teams were instructed to use the random digits tables to select distances and direction to households.

All selected households were visited from May 3–11, 2013 by a team consisting of one community health worker, village elder, interviewer, and a phlebotomist. Unoccupied homes on the first visit were revisited a single time, and households that did not agree to participate in the investigation were not replaced. After the head of household agreed to participate, all household members were asked to participate. Consenting household members were asked to complete a questionnaire that inquired about demographics, recent illness, travel history, and potential risk factors for DENV infection. In addition, one adult per consenting household also completed a questionnaire to obtain household demographics, recent history of sick household members, house construction, use of windows and/or screens, and presence of potential mosquito breeding containers in their yard. Religion was asked due to differences in clothing body coverage between the different religious groups. Responses to the household and individual questionnaires were linked to each participating individual for data analysis. All household members were asked to provide a 10 mL venous blood specimen which was allowed to clot in cold-pack refrigerated field containers; serum was separated in a Mombasa field laboratory within 4 hours of collection, stored and transported at 4°C to KEMRI where it was then stored at -80°C until tested.

### Survey Analysis

Post hoc weighting of individuals within households was performed if all individuals within a household did not participate in the survey. Confidence intervals (CI) accounted for sampling design and finite population correction factors. Logistic regression was used to assess risk factors for DENV infection, and resulting inferences accounted for the sampling design. Tests were performed with an overall Type I error of α = 0.05. All analyses were performed in R v2.15.1.

### Laboratory Diagnosis

All specimens for both hospital surveillance and the seroincidence survey were tested by enzyme linked immunosorbent assay (ELISA) to detect IgM anti-DENV antibodies (InBios International, Inc., Seattle, WA) [19–21], and by a previously described multiplex DENV-type specific, real-time RT-PCR assay to detect DENV nucleic acid [22–24]. A current DENV infection was defined by detection of DENV nucleic acid by RT-PCR. A positive IgM anti-DENV ELISA result was defined by an index value >2.84 and was considered evidence of a recent DENV infection. An IgM ELISA result between 1.65 and 2.84 was indeterminate. All indeterminates were retested, and if it was indeterminate upon repeat testing, it was classified as negative.

### Ethical Review

The investigation protocol was reviewed by human subjects review experts from the Institutional Review Boards (IRB) at CDC and KEMRI. CDC review determined the activity was consistent with a non-research public health response. A full IRB review of the protocol was required by KEMRI and approval was obtained. Informed written consent for survey participation and blood collection was obtained from all adult participants 18 years of age and older. Verbal assent and written consent from parents/guardians was obtained from minors (children 6–17 years old) and written consent from parents was obtained for children ≤ 5 years.

## Results

**Fig 1:**
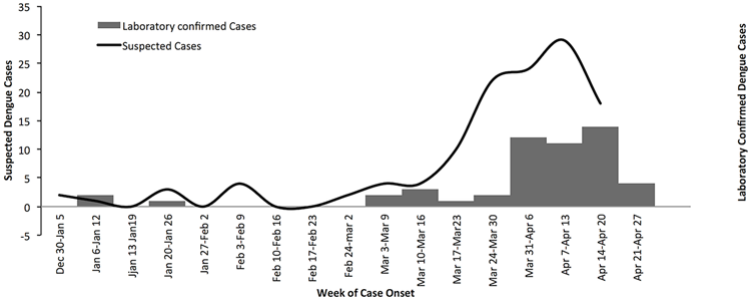
Epidemiological curve of 267 suspected dengue cases detected at hospitals from January–May, 2013, of which 155 (58%) were confirmed to have a current DENV infection and included a single fatal case.

A total of 267 suspected dengue cases were detected at hospitals from January–May, 2013, of which 155 (58%) were confirmed to have a current DENV infection and included a single fatal case. The RT-PCR positive cases ranged in age from 3 to 75 years and all cases were residents of coastal areas of Mombasa. Identified DENV types were DENV-1, DENV-2, and DENV-3 (Fig 1).

**Fig 2:**
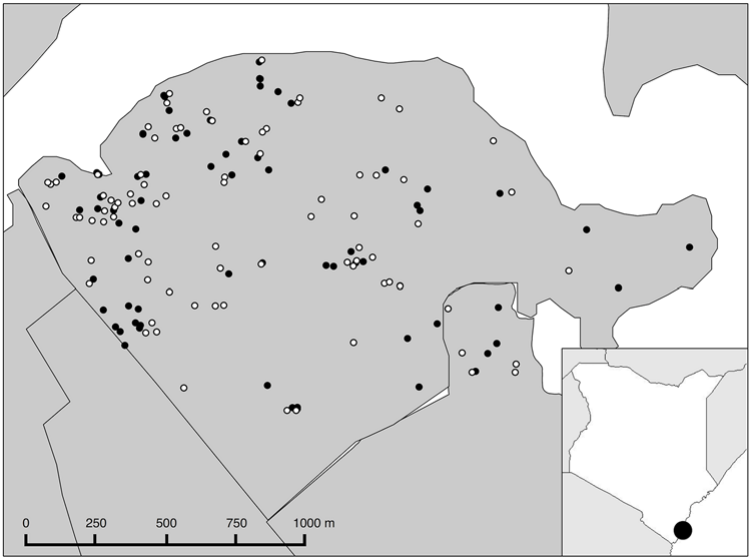
Map of 210 dengue virus (DENV) infected participants in Tudor, Mombasa, Kenya. Participants with evidence of current or recent DENV infection were distributed throughout the Tudor district, and there was no statistically significant clustering by area. Solid black circles represent IgM anti-DENV positive participants, white circles represent RT-PCR positive participants.

Of 986 randomly-selected Tudor households, 701 (71%) households including 1,500 individuals participated in the seroincidence survey (median = 2 participants per household [range: 1–11]). Median age of study participants was 28 years (range: 0.1–94), and most were Christian (67%) and female (60%), and thus similar to Mombasa’s population [5]. In total, 210 (13%; 95% confidence interval [CI] = 10–16%) participants had evidence of DENV infection with 101 current infections, including 12 participants who were both RT-PCR and IgM positive, and 109 recent DENV infections. Of the 101 RT-PCR positive participants, DENV-1 and -2 were detected in 51 (50%) and 48 (48%) of all cases, respectively, and two (2%) DENV-1 and DENV-2 co-infections were detected. Participants with evidence of current or recent DENV infection were distributed throughout the Tudor district, and there was no statistically significant clustering by area (Fig 2). There was no significant difference in infection rate between study areas within the Tudor district.

The median age of the 210 DENV infected participants was 27 years (range: 2–86), the majority were female (60%), and Christian (66%). Nearly half, 44% (95% CI = 39%–57%) of the 210 infected participants recalled having a fever in the 30 days before being interviewed, and 68 (73%) of these febrile participants reported being seen by a clinician for their illness. Approximately half (47%) of these 68 participants reported receiving a diagnosis, and all but one reported being diagnosed by their doctor with malaria while one of the patients was correctly diagnosed with dengue. Three (4%) febrile infected participants were hospitalized, and two (1%) reportedly had bleeding manifestations.

Participants with current or recent DENV infection were nearly three times more likely to report fever in the 30 days before being interviewed than non-infected participants (OR = 2.8; CI = 1.9–4.2). Participants that reported bruising were more likely to have a current or recent DENV infection (OR = 6.3; CI = 1.4–33.0) (Table 1). No other signs or symptoms were significantly associated with having a current or recent DENV infection.

**Table 1:** Demographic characteristics, symptoms and medical outcomes reported by 1,500 residents of Tudor, Mombasa, participating in serologic survey for dengue virus infection (DENV), April through May 2013.

	DENV Infection (N = 210)	No DENV Infection (N = 1,290)	OR (95% CI)
**Demographic characteristics**			
Age in years, median (range)	27 (2–86)	28 (0.1–94)	NA
Male sex, no. (%)	84 (41%)	520 (40%)	1.0 (0.7–1.8)
Religion (Christian), no. (%)	150 (71%)	876 (66%)	1.3 (0.8–2.1)
**Dengue signs or symptoms**			
Fever	93 (44%)	296 (23%)	2.8 (1.9–4.2)
Dizziness/fainting	25 (12%)	63 (4.9%)	1.4 (0.7–2.8)
Persistent vomiting	17 (8%)	33 (16%)	1.7 (0.6–4.5)
Persistent abdominal pain	25 (12%)	74 (5.7%)	0.8 (0.4–1.7)
Petechiae	3 (1.4%)	12 (1%)	1.9 (0.3–10)
Bruising	5 (2.4%)	6 (4.7%)	6.3 (1.4–33.3)
Any blood manifestation*	2 (1%)	10 (1%)	0.5 (0.1–2.4)
**Medical outcome**			
Hospitalized	3 (1.4%)	10 (1%)	0.8 (0.2–2.2)

* Bleeding manifestations included nose bleeding, bleeding from gums, blood in vomitus, blood in urine, blood in stool, or heavy vaginal bleeding.

Risk factors significantly associated with having evidence of current or recent DENV infection included report of having windows open at night (OR = 2.3; CI = 1.1–4.8), travel outside of Kenya in the past month (OR = 2.5; CI = 1.1–5.4), and failure to use mosquito repellent daily (OR = 1.6; CI = 1.0–2.8) (Table 2). Of those 12 participants who reported travel outside of Kenya in the month before being interviewed, 8/12 (73%) reported their travel destination and 7/8 (88%) had traveled to Tanzania, an area experiencing a current dengue outbreak. Of 701 participating households, the most common self-reported water containers that could serve as mosquito breeding sites in the yard were buckets (47%), septic tanks (40%), and water cisterns (31%).

**Table 2:** Risk factors associated with dengue virus infections (DENV) among residents of Tudor, Mombasa, Kenya, May 2013.

Practices	DENV Infection (N = 210)	No DENV Infection (N = 1,290)	OR (95% CI)**
	Number (%)*		
Did not use mosquito repellent daily	172 (87%)	1,038 (82%)	1.6 (1.0–2.8)
Had open windows at night	4(3%)	15 (1%)	2.3 (1.1–4.8)
Traveled outside of Kenya last 3 months	12 (10%)	58 (4%)	2.5 (1.1–5.4)
Used bed net	146 (71%)	894 (69%)	0.9 (0.6–1.6)
Had screens on windows	50 (19%)	294 (20%)	1.1 (0.5–2.2)
Kept windows open	169 (93%)	1,117 (94%)	1.1 (0.6–1.9)
Had sick household member last month	87 (35%)	477 (32%)	1.1 (0.7–1.9)
Used air conditioning	32 (20%)	190 (18%)	1.1 (0.6–2.1)
Used mosquito coils	32 (14%)	278 (25%)	0.5 (0.2–0.9)
Had breeding containers in yard	166 (84%)	1,094 (88%)	0.7 (0.4–1.2)
Had one story vs. multi-story home	57 (36%)	393 (37%)	0.7 (0.4–1.3)
Home construction permanent vs. temporary	157 (78%)	1,009 (83%)	0.7 (0.5–1.2)

* Weighted percentages are reported, reflecting the stratified sampling design. Responses were weighted to account for the different probabilities of household inclusion across strata, within-household participation rates, and inter-household clustering of infections.

** Significance level, p = 0.05. Weighted logistic regression models were used to assess risk factors for recent or current infections, and CIs were based on the modeling accounted for the sampling design. Breeding containers queried included potted plants, vegetation, wells, septic tanks, trash, buckets, water cisterns, fountains, old tires, water storage tank without lids.

## Discussion

Our survey estimated that 13% of residents of Tudor Mombasa, Kenya were infected with DENV during the 2013 dengue outbreak, or 13,782 DENV infections per 100,000 residents. Of those infected, 44% recalled having a recent acute febrile illness. While few participants reportedly required hospitalization, the majority of febrile DENV infected participants were sick enough to utilize outpatient healthcare services. Importantly, our survey found evidence of under recognition of dengue among the febrile, DENV infected participants who sought outpatient care. Similar to previous studies that found malaria being overdiagnosed in patients with acute febrile illness [25–29], we found that among participants who could recall their diagnosis, all but one reported being diagnosed with malaria. While some of our DENV-infected participants could have been co-infected with malaria, previous studies have found the incidence of malaria/DENV co-infections to be about 3% [30–32].

In our survey, the majority of febrile DENV infected participants may have been misdiagnosed as having malaria. This is important because not only is clinician awareness essential to successful disease surveillance, but early clinical recognition of dengue allows the clinician to offer accurate anticipatory guidance and timely referral for lifesaving supportive inpatient care. These secondary prevention measures have been shown to reduce medical complications and mortality among severe dengue patients from 10 to <1% [4,9,10].

Little information is available regarding the incidence of dengue in Kenya as only one comparable seroincidence survey has been conducted during an ongoing outbreak, and disease surveillance has been limited [11–18]. Our attack rate was similar to that found (14% IgM anti-DENV positive) during a dengue outbreak affecting refugee camps in neighboring Somalia almost 10 years earlier [33], and comparable to the rate of recent dengue infection during an outbreak investigation in Haiti which used the same methods [34]. The only other serosurvey was conducted in western Kenya using banked, paired serum samples from 354 afebrile children aged 12–47 months [12], which identified three anti-DENV IgG seroconversions in ~1 year period for an estimated incidence of 850 DENV infections per 100,000 persons. Regardless of the paucity of dengue incidence data, there is some evidence of dengue endemicity in at least the coastal areas of Kenya. Recent seroprevalence studies conducted during non-outbreak periods have found that ~34–53% of residents of coastal areas have evidence of prior DENV infection despite the absence of any reported outbreaks [12]. In comparison, only 1–8% of residents in non-coastal areas of Kenya have evidence of prior DENV infection; however these studies were conducted in the 1980-90s [17,18].

In areas where dengue surveillance does not exist, conducting seroincidence surveys to determine the prevalence of incident DENV infections during an apparent outbreak can be used to estimate attack rates, the extent of the outbreak and the burden of dengue [35,36]. Such an approach is now feasible because a single serum specimen can be tested by RT-PCR to detect a current DENV infection and by IgM anti-DENV ELISA to detect a recent DENV infection. This is in contrast to testing acute and convalescent specimens for IgM anti-DENV seroconversion, as was done in the past. In this outbreak, the serologic survey for incident DENV infections is especially useful for obtaining a “snapshot” of its magnitude. Recent experience using this method has shown that when combined with interview information, such surveys can be used to differentiate individuals with symptomatic or asymptomatic DENV infection, elucidate health care-seeking behaviors to estimate the degree of misdiagnosis, and identify risk factors for DENV infection. Although such serosurveys must be carefully planned to ensure representativeness of the population in question, when appropriately conducted they appear to be powerful tools to better understand and plan for the response to dengue outbreaks in areas with limited surveillance capacity.

Several risk factors for DENV infection were identified during this investigation. First, having open windows at night was a plausible risk factor because female *Aedes aegypti* mosquitoes have been shown to enter houses at night to find resting sites, and then they may take a blood meal from household members the following morning [37–39]. Second, travel outside of Kenya, specifically to Tanzania where a simultaneous dengue outbreak was occurring, may have enabled importation of DENV into Mombasa and secondary cases. This observation indicates that even in dengue endemic areas, travel to other endemic areas may constitute a risk factor for infection and highlights the need for that prevention message [40]. Lastly, daily use of mosquito repellent was found to be protective against DENV infection. Education campaigns should encourage residents to use mosquito repellent and other mosquito avoidance strategies including wearing long sleeves and pants and using permethrin-impregnated clothing.

Our investigation had several limitations. First, we made several assumptions as to the representativeness of the Tudor district when the site was selected for the seroincidence survey. Tudor district was chosen primarily because of its socioeconomic and demographic diversity, however it was also chosen because it was one of the most logistically accessible sites. Second, we were only able to estimate the incidence of DENV infection for a 90-day period prior to specimen collection based on our testing methods, which likely underestimated the true incidence of infection over the duration of the outbreak. Lastly, because there was no dengue surveillance before the outbreak and only limited surveillance after the seroincidence survey was conducted, it is unclear whether our estimates represented the beginning, peak or end of the outbreak and we were unable to directly compare these data to previous outbreaks.

Our investigation provides additional support for high level DENV transmission in coastal Kenya and important evidence for the under recognition and misclassification of dengue cases. One of the strengths of our survey to detect incident DENV infections was that it was conducted immediately following the declaration of the outbreak, which may have allowed for better recall and higher participation rates. While this outbreak illustrates the need for establishing on-going dengue surveillance to detect outbreaks, there is also the need to use ongoing dengue surveillance to describe the epidemiology of dengue in East Africa.

## Supporting Information

S1 Checklist STROBE checklist. (DOC)
